# Spiritual practices predict migration behavior

**DOI:** 10.1038/s41598-023-39587-4

**Published:** 2023-08-02

**Authors:** Daniel Auer, Johanna Gereke, Max Schaub

**Affiliations:** 1 Collegio Carlo Alberto, Political Sciences, Piazza Vincenzo Arabello 8, 10122 Turin, Italy; 2 Mannheim Centre for European Social Research MZES, University of Mannheim, A5/6, Building A/B, 68159 Mannheim, Germany; 3 Johannes Gutenberg University Mainz, Institute for Sociology, Jakob-Welder-Weg 12, 55128 Mainz, Germany; 4 University of Hamburg, Department of Political Science, Allende-Platz 1, 20146 Hamburg, Germany; 5 WZB Berlin Social Science Center, Reichpietschufer 50, 10785 Berlin, Germany

**Keywords:** Environmental social sciences, Psychology and behaviour

## Abstract

Each year, several thousand migrants from sub-Saharan Africa lose their lives attempting to reach Europe’s southern shores. Social scientists and policymakers have puzzled over the question of why so many people are willing to take this extremely high risk of dying. Drawing on panel data from over 10,000 individuals collected over the course of 1 year in The Gambia—a country with one of the highest emigration rates in the world—we show that consulting a local healer for spiritual protection predicts migration outcomes. Furthermore, we find that spiritual practices are strongly associated with a decreased perception of one's own risk of dying on the migration journey. Our findings demonstrate the relevance of ideational factors in explaining risky migration choices, and point to spiritual leaders as important interlocutors for migration policy makers.

## Introduction

The International Organization for Migration (IOM) estimates that between 2014 and 2023 approximately 28,000 people have died trying to cross the Mediterranean Sea from North Africa to Europe, making this the deadliest route worldwide. As the number of migrants attempting to cross the Mediterranean has increased again since 2021, so too have deaths at sea (see IOM Missing Migrants recorded in the Mediterranean since 2014 https://missingmigrants.iom.int/region/mediterranean and IOM Flow Monitoring—MediterraneanArrivals https://dtm.iom.int/europe). Among those taking these dangerous journeys are many young people from sub-Saharan Africa who are fleeing poverty, repressive governments, and conflicts in search for a better future in Europe.

In response, the European Union, several European governments, institutions and inter-national organizations have launched information campaigns designed to discourage aspiring migrants from making the dangerous journey by raising awareness of the potential risks of irregular migration^1–6^ (there is no agreed upon definition of irregular migration but migrants crossing international borders outside legal channels, such as arriving without a valid visa, are usually considered irregular^7^). Behind these efforts is the assumption that aspiring migrants lack important information about the actual risks of irregular migration across the Mediterranean Sea.

Social scientists studying migration have long pointed to risk and uncertainty as important factors characterizing migration decision-making^8–12^. However, existing migration theories trying to explain why some people migrate while others stay, have largely focused on socioeconomic and structural factors^13,14^. In contrast—and despite their acknowledged theoretical importance—perceptions of risk and uncertainty have so far received relatively little attention in the empirical literature on migrant decision-making^9,15^. Qualitative evidence on irregular migration suggests that migrants are often aware of the high risks^16–19^ but that this information appears to be largely irrelevant to their migration decisions^20^. In other words, migrants “are willing to participate in the lottery called Europe,” hoping that they will be successful at making the so-called “backway” journey to Europe via the Mediterranean Sea^21^. This raises the question why, despite their awareness, they still decide to dare the journey.

Ethnographic accounts have documented the ubiquity of supernatural and religious beliefs and spiritual practices as risk management strategies of aspiring migrants in such high-risk migration contexts^16,22–24^. For example, research in Senegal has documented how religious guides (marabouts) provide migrants with a range of services, from spiritual protection, to suggesting appropriate departure dates, to scanning passenger lists for problematic individuals with whom contact should be avoided^16^. Other research has found that (Muslim) Ghanaian migrants travelling irregularly towards Europe often deliberately travel during Ramadan due to the belief that the risks might be smaller^21^. However, there is little quantitative empirical work that would allow us to estimate the extent to which such beliefs and practices are present and to assess the extent to which they predict migration behavior.

Findings from the African Religion Survey (2010) by the Pew Research Center’s Forum on Religion & Public Life with approximately 25,000 respondents in 19 sub-Saharan countries (though not including The Gambia) point to the prevalence of supernatural beliefs and practices in sub-Saharan Africa. The survey found that 39% of respondents believed that spiritual people can protect themselves from bad things happening, approximately 26% said they believe in the protective power of jujus (charms or amulets), and 40% indicated that they or their family attend services of traditional religious healers^25^.

We extend this work by providing, for the first time, quantitative evidence of the link between magical beliefs, risk perceptions (in particular the risk of dying during the migration journey) and migration attempts using large-scale panel data (N = 10, 181) collected among young adults in The Gambia, a country with one of the highest migration rates in the world. We argue that magical beliefs are associated with a higher likelihood of attempting to migrate (mostly irregularly in the case of The Gambia where regular migration channels to Europe are almost nonexistent), and that the underlying mechanism is superstition helping aspiring migrants to manage the uncertainty inherently associated with migration.

Our study also contributes to the broader literature on individual-level determinants of (irregular) migration by highlighting how ideational factors, such as practices rooted in supernatural beliefs, influence risk perceptions and subsequent migration behavior. So far, most scholars taking a micro-perspective have focused on socioeconomic incentives in explaining individual decisions to migrate^8,26^. However, beliefs and norms can also shape both (a) the value that individuals attach to different choices, and (b) the cognitive mechanisms they employ to decide between different options. We add to this general line of research by demonstrating the influence of culturally-specific spiritual practices on risk perceptions and migration outcomes^27^.

We also contribute to the literature on the relationship between magical thinking and religious and spiritual practices. Extant research has shown that magical and religious beliefs are associated with reduced risk perceptions and reduced anxiety, but that beliefs only strongly affect behavior when combined with ritual practice^28,29^. For example, a study from Mauritius showed how partaking in a religious ritual led to a reduction in anxiety among individuals while there was no such effect among members of a control group holding similar beliefs who did not take part^30^. Our findings add to this literature by showing that engaging in spiritual practices is associated with both a higher perceived chance of surviving the migration journey and more frequent migration attempts.

We find that personal knowledge about someone who has died attempting to migrate on the “backway” to Europe is widespread in The Gambia (76%) but that aspiring migrants are nevertheless often optimistic that they themselves will survive the journey. This optimism is particularly widespread among individuals who seek spiritual protection from local healers (around 30% of our sample) and those who wear jujus (protective amulets, 14%, Table S1). Using the panel structure of our data, we show that consulting a local healer, such as a Sheikh (religious healer) or Billewo/Timowo (traditional healers) is associated with one’s subsequent migration behavior. Individuals who engage in such spiritual practices are 5 to 10 percentage points (pp) more likely to undertake a migration attempt in the subsequent time period.

We do not argue that individuals are fundamentally irrational, or that their behavior is erratic and non-predictable. Rather, we believe that human beings are purposeful actors who have goals in mind, and whose behavior is geared towards the realization of preferred outcomes. However, we also acknowledge that aspiring migrants face great uncertainty when trying to map behavior onto outcomes. For example, they may wonder “if I migrate, will I successfully make the passage across the Mediterranean Sea, or will I be left destitute and stranded in transit?” Supernatural beliefs may help migrants navigate this intrinsic uncertainty by making sense of randomness and providing them with a sense of control (external locus of control)^31^. These beliefs may also have other psychological benefits, such as reduced anxiety and depression^32,33^.

From a methodological point of view, this study allows for a rare analysis of actual migration behavior rather than relying solely on intentions and/or plans, as is common in recent empirical studies on risk perceptions and (irregular) migration^12^. This is made possible due to the large sample size and the study’s three wave panel structure, where respondents were followed over the course of almost a year.

Our results call for more research on the link between supernatural beliefs and practices and aspiring migrants’ risk perceptions. The study also has implications for policy makers and activists seeking to influence aspiring migrants in their decision to attempt the dangerous journey from Africa to Europe. Information campaigns typically appeal to migrants’ rational calculus by highlighting the risks of irregular migration and have been shown to influence migration intentions (net of sociodemographic and socioeconomic factors) in some contexts^12^. However, our results point out the limits of such information campaigns given that migrants are willing to take the risks as long as they perceive themselves to have been “blessed with spiritual protection” to succeed.

## Results

This section presents the main results visually. The SI Appendix provides further details regarding the statistical models as well as auxiliary analyses (see S3 and S4). Our main hypothesis and test were pre-specified and pre-registered prior to obtaining the data at OSF (https://osf.io/4thfk). Receiving a magic spell from a spiritual healer is positively associated with individuals’ migration propensity. We first examine whether going to a spiritual healer to receive a protective spell is associated with individuals’ international migration propensity. We assess migration propensity with the question—asked both in wave 2 and wave 3—“Since we last talked, have you left your home to live in another country but returned?”. We further clarified that “left to live abroad” means that the respondent had intended to stay abroad for at least three months, explicitly excluding short visits or business trips. If respondents could not be reached personally via their cell phone, we contacted their “representatives” and asked them if the respondent was currently abroad.

Our main independent variable is whether respondents recently received a magic spell or juju/gri-gri from a spiritual healer to protect or strengthen them, which we asked in all waves (We differentiated among Sheikhs (religious healers) and Billewos/Timewos (traditional healers) in the survey but pool the different types of healer in the main analysis because we have no theoretical reason to examine differences across them. Results look very similar when conducting the analysis separately for the different types of healer. See Fig. S2). A juju/gri-gri is a talisman worn to protect the wearer from evil and to bring luck. Our analysis demonstrates a strong association between receiving a protective spell from a healer and international migration attempts both when using the cross-sectional model (with and without sociodemographic controls and controlling for previous migration experience and migration intentions) as well as when we follow respondents in the panel over time using individual level fixed effects, which help to rule out unobservable confounders.

**Figure 1:**
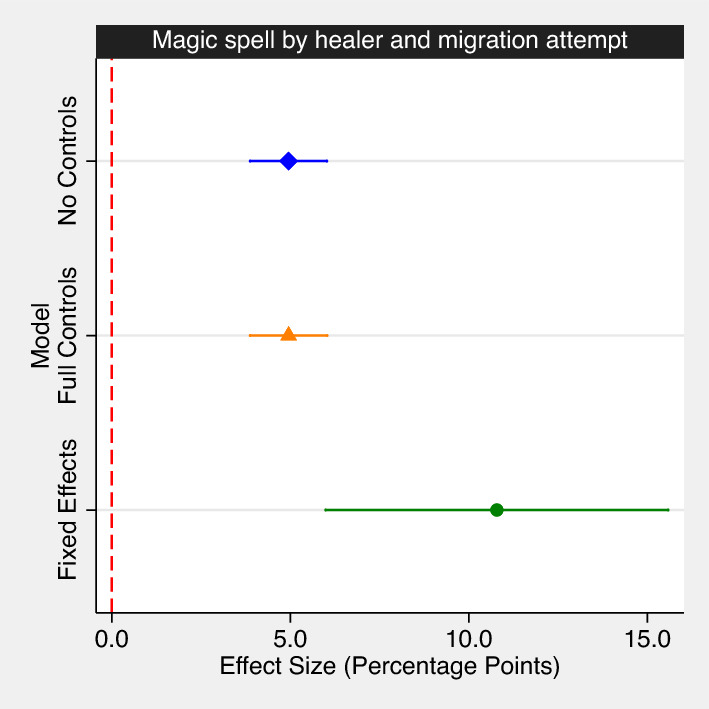
Magic spell by healer and migration attempt. The coefficient plot presents the predicted effect of receiving a protective spell from a spiritual healer on international migration attempts. Marginal effects after logistic regression. (1) Cross-sectional, multilevel model with intercepts varying at the level of the individual but without controls, (2) cross-sectional, multilevel model including the full set of sociodemographic controls, and (3) longitudinal analysis including respondent fixed-effects. Markers are point estimates, bars show 95% confidence intervals. Full regression results available in Tables S2 and S3.

As shown by the predicted marginal effects in Fig. 1, this difference is statistically significant (p < 0.01) in all model specifications. When respondents report having recently received a protective spell from a healer, their predicted probability of going abroad increases by 5 pp from around 10% to 15%. This effect is sizable and, for instance, twice as large as the association between male gender and migration propensity. The results are even stronger in the FE (within-person) model, where having received a magic spell from a healer is associated with a substantial 10 pp increase in the predicted probability of going abroad. Our results are robust to restricting the sample to respondents who themselves answered the survey rather than their family members or friends (see Models 2 and 4 in Table S3). Neither are our results biased by attrition, as we show in the robustness checks (Table S6) where we restrict our pooled cross-sectional analysis to those individuals who were present and answered the survey themselves in both wave 2 (N = 7, 737) and wave 3 (N = 6, 430). We observe similar results when using the wearing of a juju, i.e., a protective amulet or talisman, as an alternative independent variable. This is a behavioral measure: Enumerators recorded in the initial interview if the respondent reported wearing a juju (14%) and if it was shown to the enumerator (3 out of 4 of those respondents wore their amulet or talisman visibly). The positive association between a third person (the enumerator) seeing the juju and the respondent’s subsequent migration propensity adds robustness to our main findings (see Table S7). Holding all individual attributes constant, both receiving a protective spell and wearing an amulet systematically predict subsequent migration.

We do not argue that receiving a magic spell in itself *causes* individuals to undertake migration journeys but rather think of partaking in spiritual practices as lying on the causal path running from spiritual and religious beliefs to migration (see Fig. S3 for a directed acyclic graph (DAG) illustrating the hypothesized causal process). This said, seeking spiritual protection does appear to be an integral part of the migration process. An additional test provides further evidence for this claim. As shown by van Bemmel^21^, it is believed that Ramadan, the holy month of Islam, provides an elevated level of protection for Muslims seeking to migrate. This should reduce the need for spiritual protection through (costly) jujus/grigris. In our models, we would hence expect the interaction between the interview taking place during Ramadan and seeking spiritual protection to be negative, which is indeed what we find (see Table S8).

We theorized that seeking spiritual protection likely serves as a risk-management strategy. When we examine how aspiring migrants evaluate their own risk of dying during the journey from West Africa to Europe, we find that respondents are fairly uncertain about their own survival. Our measure of risk asks respondents to rate how likely they are to survive the journey should they decide to migrate to Europe on the “backway” (i.e., migrate irregularly) on a scale from 0 to 100, where 0 means that they would not survive, and 100 means that they would certainly survive. On average, the respondents estimate the probability of own survival at 50%. Strikingly, this—rather pessimistic—estimate does not significantly differ among respondents with and without prior migration experience, or those with and without concrete migration plans, illustrating just how risky this type of migration is being perceived.

**Figure 2:**
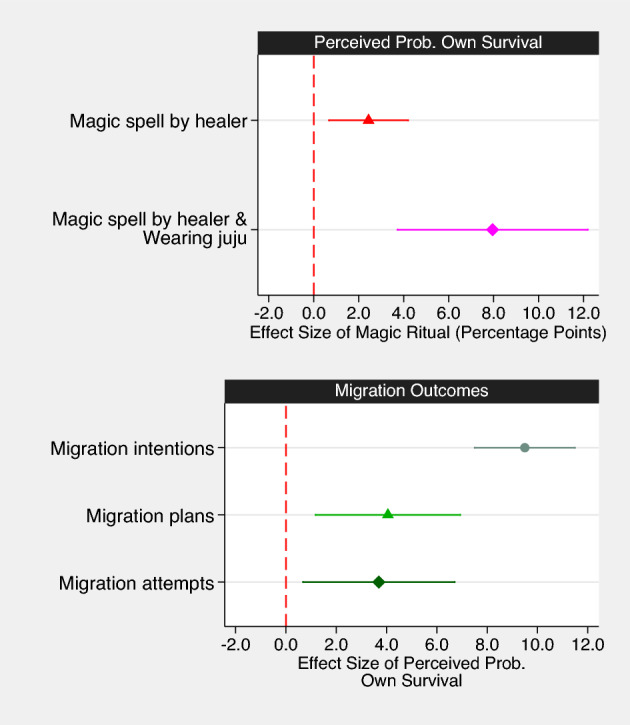
Mechanism. The coefficient plot in the top panel shows the estimated effects of receiving a protective spell from a healer and wearing a juju on the self-assessed likelihood to survive the migration journey across the Mediterranean Sea. Markers are point estimates, bars represent 95% confidence intervals. Marginal effects after OLS regression controlling for the full set of sociodemographic characteristics. Full results are shown in Table S4. This figure is based on the survey results from wave 1 (Model 4). Individuals who have received a magic spell from a healer and wear a juju perceive their chances of survival as significantly greater than the average. The bottom panel shows the estimated effects of the self-assessed likelihood to survive the migration journey across the Mediterranean Sea on respondents’ migration intentions, plans, and attempts. Full estimation results are available in Table S5. This figure is based on the survey results of migration intentions and concrete migration plans (wave 1), and migration attempts (pooled from wave 2 and 3). Individuals who perceive their chances of survival as greater than the average intend to go abroad, hold concrete migration plans, and make actual migration attempts at significantly higher rates.

However, we find that individuals who engage in supernatural practices (i.e., seek protective spells from a healer or wear a juju) are significantly more optimistic about their own probability of surviving. As shown in Fig. 2, the assessed probability of own survival is about 2 pp higher amongst respondents who have visited a healer. For respondents who have both visited a healer and wear a juju, the predicted probability is about 58%, i.e., 8 pp higher than average (see Table S4 for full results). The survey data also reveal that a lower perceived risk of dying on the backway to Europe is positively associated with migration intentions, migration plans, and migration attempts (see Fig. 2 and Table S5). Compared to an individual who does not believe s/he would survive the journey, a person who believes that s/he would definitely survive the journey is 10 pp more likely to indicate an intention to migrate (baseline intention to migrate: 65%, Model 1), 4 pp more likely to have migration plans (baseline likelihood of having plans: 57%, Model 4), and 4 pp more likely to make a migration attempt (baseline likelihood: 20%, Model 7). These additional results suggest that spiritual protection can indeed function to rationalize risky behavior and spur migration.

## Discussion

Migration—as most human behavior—is tied to risk and uncertainty. Supernatural beliefs and spiritual practices can provide individuals with a sense of control in risky and potentially life-threatening situations. In this study, we investigated the role of spiritual practices as a predictor of international migration behavior. Using panel data on more than 10,000 young Gambians we show that receiving spiritual protection by a healer strongly predicts subsequent migratory behavior. We also find that individuals who engage in spiritual practices are more likely to believe that they would survive the migration journey.

To the best of our knowledge, we are the first to provide large-scale quantitative evidence for the role of spiritual practices and beliefs in the migration decision-making process. We show that ideational factors can be as influential as well-documented criteria, such as socioeconomic factors (e.g., poverty, employment or education) or policy constraints^18,34–36^. Our findings inform the discussion on the role of risk preferences for migration decisions. This literature has mostly focused on domestic migration, i.e., a setting with obviously smaller and less immediate risks^9,10,15^ or the correlational association between risk perceptions and migration intentions, rather than behavior^12,36^. We explicitly show that superstitious beliefs go along with increased optimism with regard to the probability of surviving the perilous irregular migration journey and more frequent migration attempts.

Our results also have important implications for understanding the role of gatekeepers in migration decision making. We provide robust evidence that spiritual leaders take on a crucial role when it comes to influencing the migration plans of their “clients”, confirming earlier qualitative evidence^16^. From a policy perspective, spiritual leaders, then, are important gatekeepers for migration management whose insights and influence could be drawn on to reduce deaths during the migration journeys.

## Materials and methods

### Study setting and data

We collected our data in the West African country of The Gambia, a nation that has only recently escaped a decades-long dictatorship and which is struggling with widespread poverty and a lack of economic opportunities. The latest available composite Human Development Index ranks it 174 out of 188 countries as of 2019^37^.

The agglomeration of Kanifing and Serrekunda around the capital city Banjul is the only major urbanized area in the country and home to about half of the country’s population of 2.3 million inhabitants in 2018^38^. The area attracts many young Gambians who moved from the countryside for education and (scarce) job opportunities. Consequently, the Greater Banjul area is also a main point of departure for international migrants.

In January 2019, we sent a team of 30 local enumerators to 500 randomly selected locations across the populated area in Greater Banjul. These locations were chosen in a two-step procedure: We first selected census blocks in relation to their population size, and then generated random enumerator starting points within the blocks. Once enumerators arrived at the starting points, they approached the 20 individuals closest to the point and invited them to be interviewed. The sampling frame and enumerator starting points are shown in Fig. S1.

Eligible for interview were all individuals aged between 15 and 35 years—the age group most strongly represented among migrants. Since the majority of migrants who engage in irregular migration from The Gambia tend to be men, we oversampled young men 4:1. The interview consisted of a short (10-min) survey at the end of which respondents were asked to provide detailed contact information. This information consisted of the respondent’s phone number and the phone number of a person close to the respondent who would know about the respondent’s situation and whereabouts in case we could not reach the respondent later on. The original recruitment round was followed by two follow-up waves that took place in May/June (wave 2) and September/November 2019 (wave 3). During waves 2 and 3, respondents were contacted via phone. If they could not be reached, we tried to contact their named “representatives”. This step ensured that we could still obtain information about the respondent even in case the respondent had left the country—which is crucial for tracking migration behavior. The data collection was approved by the WZB Research Ethics Review Board (Decision 2019/57) and was carried out in accordance with all relevant guidelines and regulations. We obtained informed consent from all respondents at the beginning of the first wave. We took several additional steps to tackle attrition in our panel. First and foremost, we paid an incentive to take part in each of the three waves in the form of a lottery. We also advertised the survey (and the lottery) in advance on radio shows on one of The Gambia’s largest stations. Eventually, we managed to re-contact 90% of the initial sample at least once in wave 2 and/or wave 3. Importantly, attrition was not systematically driven by respondents’ sociodemographic characteristics (see Table S1 for differences in means tests and detailed information on attrition).

Our sample is predominantly male (82%), single (79%), without children (78%) and approximately 26 years old and with 10 years of education. While we have no a priori assumptions that our findings are not generalizable to other demographic or age groups, we acknowledge existing research highlighting that with increasing age, people also change their migration propensity, risk preferences^13,39^, and religious practices. Hence, additional research is needed to test the generalizability of our results. Almost one third (30%) of the respondents had already migrated or attempted to migrate abroad prior to our first interview. Approximately 70% of all respondents indicated that they intended to move abroad. To capture migration behavior during the panel phase, we use a dichotomous measure that equaled 1 if the respondent said s/he migrated and 0 otherwise. Among our respondents, around 23% undertook an international migration journey: 14% between the first and the second wave, and 9% between the second and the third wave of our survey. More information on the composition of our sample of young Gambians is provided in Table S1.

### Statistical analysis

We use two different analytical strategies: first, we estimate the cross-sectional relationship between consulting a healer and migration behavior using the following simple specification:

1 $${M}_{i} = {\tau }_{i}+ \lambda {X{^\prime}}_{i}+{\varepsilon }_{i},$$

where the individual migration propensity M (moved abroad or attempted move abroad) is a function of attending a healer (sheikh, billowo, or timowo) and individual sociodemographic control variables (including gender, age, education, economic status, family situation, and prior migration experience; see S3 for the full model). We include the control variables to account for the fact that people who go abroad or go to healers may be different in observable ways from those who do not go abroad or do not go to a healer. This simple model provides a first informative picture of whether individuals who hold supernatural beliefs, i.e., who seek spiritual protection by healers, are potentially more likely to migrate (τ in Eq. 1).

Second, the longitudinal data structure with three waves over the course of one year enables us to estimate a more demanding individual fixed-effects model:

2 $${M}_{{i}_{t}}= \psi {H}_{{i}_{t-1}} + \lambda {X{^\prime}}_{{i}_{t-1}} +{\delta }_{i} + {\varepsilon }_{{i}_{t}},$$

where the individual migration propensity M in period t (migration or migration attempt between 1st and 2nd wave or between 2nd and 3rd wave) is a function of individual time-variant characteristics X′_it−1_ and healer attendance in the previous wave H_it−1_, and time-invariant unobserved individual factors δ_i_. The effect of healer attendance on migratory behavior is then captured by the coefficient ψ in Eq. (2).

## Supplementary Information

Supplementary Information.

## Data Availability

All data and replication code are available on OSF (https://osf.io/5bsjp/).
