# Supplementary material for: Spiritual practices predict migration behavior
Source: Sci Rep. 2023 Aug 2;13:12535. doi: 10.1038/s41598-023-39587-4 (PMC10397254; doi:10.1038/s41598-023-39587-4)
Supplement: Supplementary file 1 — Supplementary Information. [file 41598_2023_39587_MOESM1_ESM.docx]

Supplementary Materials for

**Spiritual practices predict migration behavior**

**The PDF file includes:**

Materials and Methods, S1

Figs. S1 to S3

Summary Statistics, S2

Table S1

Main Regression Results, S3

Tables S2 to S5

Robustness Checks, S4

Tables S6 to S8

S1. Materials and Methods

Sampling Frame

**Fig. S1. Sampling Frame in the Greater Banjul area, The Gambia (*N*=10,181)**

*
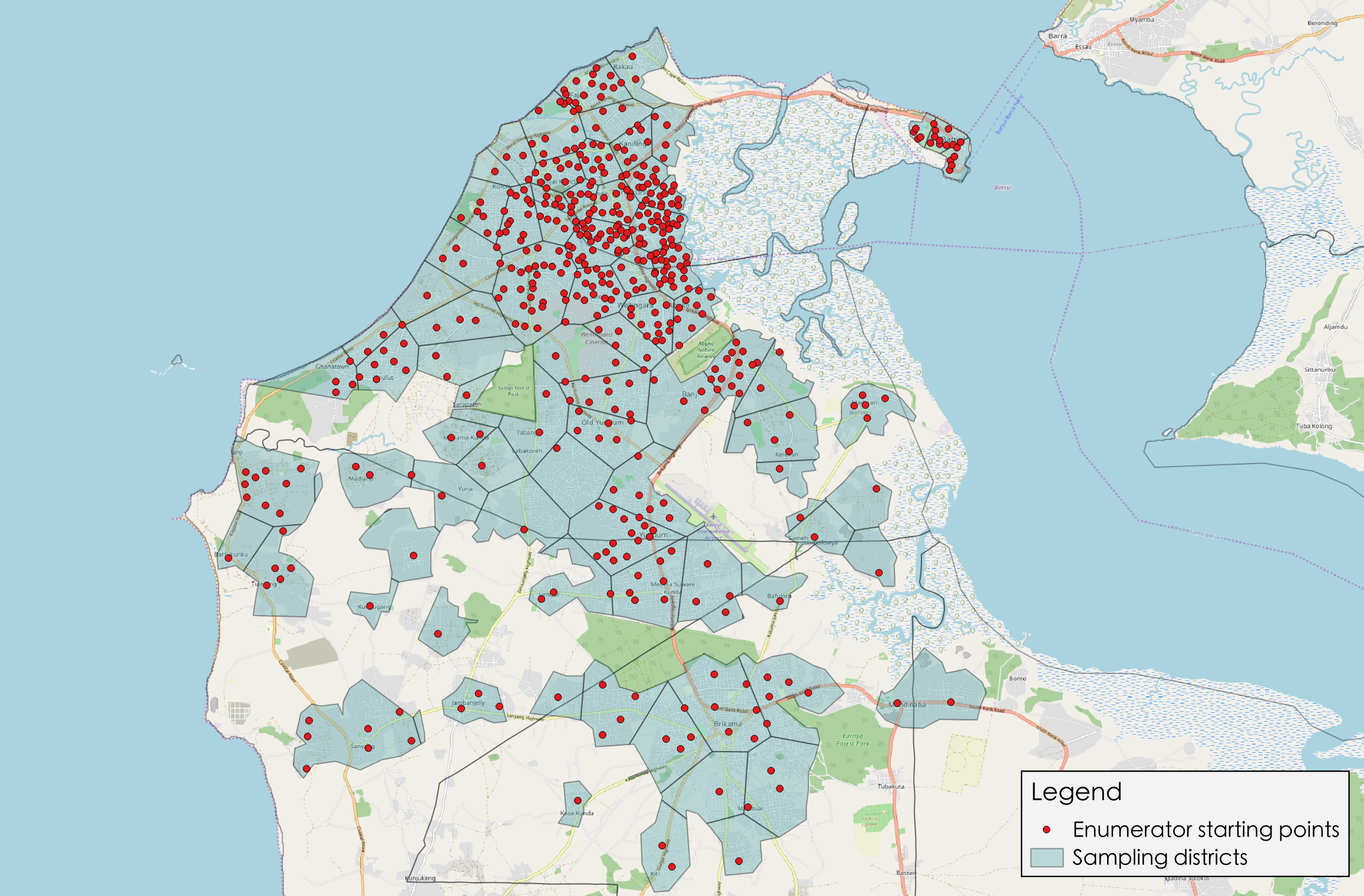
*

*Notes*: The Figure shows the sampling frame and enumerator starting points used to recruit the respondents of our survey. The sampling frame consisted of the population of the Greater Banjul area, consisting of the administrative districts of Banjul, Kanifing, Kombo Central, Kombo South, Kombo North, and the north of Brikama district. According to the 2013 population and housing census, these areas were home to 53.54% of the Gambian population. The sampling procedure consisted of two steps: In a first step, we sampled ‘settlements’ – disaggregated spatial units of around 6,000 individuals, for which detailed information on the population numbers and composition are available ([1](#_bookmark2)). From the Gambia Bureau of Statistics we obtained the coordinates of the settlement centroids. We approximated the settlements extents by creating Voronoi diagrams around the centroids. We then drew a weighted random sample of settlements, setting the settlement population of 15-49 year-olds – the census age-category closest to our 15-35 year target group – as weight. In a second step, we assigned enumerator starting points. We randomly dropped 500 points – again with the number of starting points relative to the local young adult population – and snapped them to the road network. Enumerators were then instructed to recruit 20 individuals in the vicinity of each starting point. Figure created by one of the authors (M.S.) using [QGIS 3.2.3-1](https://www.kyngchaos.com/files/software/qgis/QGIS-macOS-3.2.3-1.dmg) (archived at https://www.kyngchaos.com/software/archive/qgis/).

Fig. S2. Magic spell by healer and migration attempt

**

*Notes*: The coefficient plots present a comparison of the association between going to different types of spiritual healers and international migration attempts of survey respondents. The first panel shows the OLS regression estimates from the cross-sectional analysis without controls. The second panel shows the OLS coefficients of the cross-sectional analysis with full socio-demographic controls. The third panel shows the longitudinal analysis using OLS with respondent fixed effects. Bars show 95% confidence intervals. Full sample is shown.

Fig. S3. Directed acyclic graph showing hypothesized causal links

*Notes*: Hypothesized causal links between spiritual beliefs, part-taking in spiritual/religious rituals, and migration attempts. The arrows indicate hypothesized causal links, the t-indices show the assumed temporal order.

# S2. Summary Statistics and information on attrition

Table S1. Respondent characteristics across three panel waves

| (1) | | (2) | (3) | | (4) | | (5) | |  |
| --- | --- | --- | --- | --- | --- | --- | --- | --- | --- |
|  | Wave 1 | Wave 2 | Wave 3 ∆1 *−* 2 ∆1 *−* 3 | | | | | | |
| *Reported in wave 1* |  |  |  |  | |  | |  |  |
| Male | 0.815 | 0.817 | 0.820 | 0.002 | | 0.005 | |  |  |
|  | (0.004) | (0.004) | (0.005) | (0.006) | | (0.006) | |  |  |
| Age | 25.946 | 26.002 | 25.971 | 0.056 | | 0.025 | |  |  |
|  | (0.059) | (0.065) | (0.071) | (0.088) | | (0.092) | |  |  |
| Single | 0.699 | 0.703 | 0.699 | 0.005 | | 0.000 | |  |  |
|  | (0.005) | (0.005) | (0.005) | (0.007) | | (0.007) | |  |  |
| No children | 0.784 | 0.784 | 0.789 | -0.001 | | 0.005 | |  |  |
|  | (0.004) | (0.004) | (0.005) | (0.006) | | (0.007) | |  |  |
| Years of education | 9.628 | 9.757 | 9.865 | 0.128* | | 0.236*** | |  |  |
|  | (0.041) | (0.045) | (0.048) | (0.061) | | (0.065) | |  |  |
| Income (1000 GMD, group) | 1.550 | 1.569 | 1.568 | 0.019 | | 0.017 | |  |  |
|  | (0.023) | (0.026) | (0.028) | (0.035) | | (0.037) | |  |  |
| Meals skipped per week | 0.335 | 0.331 | 0.325 | -0.004 | | -0.007 | |  |  |
|  | (0.005) | (0.005) | (0.006) | (0.007) | | (0.007) | |  |  |
| Previous migration | 0.298 | 0.295 | 0.285 | -0.003 | | -0.013* | |  |  |
|  | (0.005) | (0.005) | (0.005) | (0.007) | | (0.007) | |  |  |
| Knows sb. who died mig. | 0.757 | 0.756 | 0.758 | -0.001 | | 0.002 | |  |  |
|  | (0.004) | (0.005) | (0.005) | (0.006) | | (0.007) | |  |  |
| Wears juju | 0.142 | 0.137 | 0.136 | -0.005 | | -0.006 | |  |  |
|  | (0.003) | (0.004) | (0.004) | (0.005) | | (0.005) | |  |  |
| *Reported in multiple waves* |  |  |  |  | |  | |  |  |
| Recent magic spell by healer | 0.293 | 0.274 | 0.230 | -0.019*** | | -0.063*** | |  |  |
|  | (0.005) | (0.005) | (0.005) | (0.007) | | (0.007) | |  |  |
| Migration intention | 0.693 | 0.717 | 0.742 | 0.024*** | | 0.048*** | |  |  |
|  | (0.003) | (0.004) | (0.004) | (0.005) | | (0.006) | |  |  |
| Willingness to take risks | 0.395 |  | 0.427 |  | | 0.033*** | |  |  |
|  | (0.004) |  | (0.005) |  | | (0.006) | |  |  |
| Migration attempt |  | 0.139 | 0.093 |  | |  | |  |  |
|  |  | (0.004) | (0.003) |  | |  | |  |  |
| *N* | 10,181 | 8,351 | 6,268 | 18,532 | | 16,449 | |  |  |
| *Attrition information* |  |  |  |  | |  | |  |  |
| N Initial sample |  |  |  |  | | 10,181 | |  |  |
| N Interviews Wave 2 |  |  |  |  | | 8,396 | |  |  |
| N Interviews Wave 3 |  |  |  |  | | 6,301 | |  |  |
| Re-interviewed in Wave 2 & Wave 3 |  |  |  |  | | 5,543 | |  |  |
| Re-interviewed in Wave 2 only |  |  |  |  | | 2,853 | |  |  |
| Re-interviewed in Wave 3 only |  |  |  |  | | 758 | |  |  |
| Share with 3 observations |  |  |  |  | | 0.544 | |  |  |
| Share with at least 2 observations |  |  |  |  | | 0.899 | |  |  |

*Notes*: The table presents descriptive statistics on respondent characteristics, changes in the composition of the sample across the different waves using ordinary least-squares (OLS) regressions with wave indicators, and the retention rate across the three different waves. Basic sociodemographics as well as information on whether respondents had any prior migration experience, knew somebody who died on the so-called “backway" migration journey from Africa to Europe, and wore a juju (talisman) were recorded in the initial wave only (N=10,181). Our main independent variable whether respondents recently received a magic spell or juju/gri-gri from a healer to protect or strengthen them was recorded in each wave. The general willingness to take risks was measured in wave 1 and wave 3. Migration attempts were recorded in wave 2 and wave 3. We were able to re-interview N=8,396 in wave 2 and N = 6,301 in wave 3. Totals that deviate from these numbers are due to missing observations in individual variables. Model 4 reports changes in the composition of the sample between wave 1 and 2. Model 5 reports changes between wave 1 and 3. The Models 4 and 5 show that attrition was not systematically driven by respondents’ sociodemographic characteristics. Significance levels: * *p <* 0*.*05,** *p <* 0*.*01, *** *p <* 0*.*001.

# S3. Main Regression Results

Table S2. Pooled Between-Person Comparison: Going to a Healer and Migration Attempt in W2 or W3

|  | (1) | (2) | (3) | (4) | (5) | (6) | (7) | (8) |
| --- | --- | --- | --- | --- | --- | --- | --- | --- |
| Magic spell by healer | 4.95*^***^* (0.01) | 4.90*^***^* (0.01) | 4.95*^***^* (0.01) | 5.00*^***^* (0.01) |  |  |  |  |
| By a sheikh |  |  |  |  | 4.58*^***^* (0.01) | 4.66*^***^* (0.01) | 4.63*^***^* (0.01) | 4.57*^***^* (0.01) |
| By a billewo/timowo |  |  |  |  | 9.85*^***^* (0.02) | 9.71*^***^* (0.02) | 9.63*^***^* (0.02) | 9.37*^***^* (0.02) |
| By sb else |  |  |  |  | 4.91*^***^* (0.01) | 4.89*^***^* (0.01) | 4.92*^***^* (0.01) | 5.09*^***^* (0.01) |
| Previous Migration Experience |  | 2.75*^***^* | 2.32*^***^* | 2.22*^***^* |  | 2.73*^***^* | 2.31*^***^* | 2.20*^***^* |
|  |  | (0.01) | (0.01) | (0.01) |  | (0.01) | (0.01) | (0.01) |
| Migration Intention |  |  | 0.02*^*^* | 0.01^+^ |  | 0.02*^*^* | 0.02*^*^* | 0.01^+^ |
|  |  |  | (0.00) | (0.00) |  | (0.00) | (0.00) | (0.00) |
| Male |  |  | 2.15*^**^* (0.01) | 2.52*^***^* (0.01) |  |  | 2.15*^**^* (0.01) | 2.53*^***^* (0.01) |
| Above 26 years |  |  | 1.52*^*^* | 1.40^+^ |  |  | 1.50*^*^* | 1.39^+^ |
|  |  |  | (0.01) | (0.01) |  |  | (0.01) | (0.01) |
| Single |  |  | 0.79 | 0.86 |  |  | 0.79 | 0.86 |
|  |  |  | (0.01) | (0.01) |  |  | (0.01) | (0.01) |
| No Children |  |  | 0.47 | 0.61 |  |  | 0.46 | 0.60 |
|  |  |  | (0.01) | (0.01) |  |  | (0.01) | (0.01) |
| Years in Education |  |  | -0.03 | -0.03 |  |  | -0.03 | -0.03 |
|  |  |  | (0.00) | (0.00) |  |  | (0.00) | (0.00) |
| Std. Income |  |  | 0.53^+^ | 0.59*^*^* |  |  | 0.53^+^ | 0.59*^*^* |
|  |  |  | (0.00) | (0.00) |  |  | (0.00) | (0.00) |
| Skipped meal last week |  |  | -0.70 | -0.77 |  |  | -0.70 | -0.78 |
|  |  |  | (0.01) | (0.01) |  |  | (0.01) | (0.01) |
| Wave 3 | -6.33*^***^* | -6.30*^***^* | -6.34*^***^* | -6.07*^***^* | -6.21*^***^* | -6.20*^***^* | -6.22*^***^* | -5.96*^***^* |
|  | (0.01) | (0.01) | (0.01) | (0.01) | (0.01) | (0.01) | (0.01) | (0.01) |
| N | 14,436 | 14,436 | 14,379 | 13,498 | 14,436 | 14,379 | 14,379 | 13,498 |
| Individuals | 9,068 | 9,068 | 9,044 | 8,744 | 9,068 | 9,044 | 9,044 | 8,744 |
| Sample | Full | Full | Full | Respondent Only | Full | Full | Full | Respondent Only |
| Random Effects | Yes | Yes | Yes | Yes | Yes | Yes | Yes | Yes |

*Notes*: Marginal effects after multilevel logistic regression with intercepts varying at the level of the individual. The dependent variable is a dummy variable taking the value 1 if the following questions were answered affirmatively, and 0 if else: “During the last 6 months, have you left your home to live in another country but returned/ Since we last talked, have you left your home to live in another country but returned?". These questions were asked in W2 and W3, respectively. The main IV recorded in all waves is “Have you recently received a magic spell or gri-gri to protect or strengthen you?". Models 1 to 4 use a pooled indicator recording whether an individual visited a healer or not, and Models 5 to 8 show results disaggregated by type of healer. Significance levels: ^+^, *p <* 0*.*10, * *p <* 0*.*05, ** *p <* 0*.*01, *** *p <* 0*.*001. Models 2 and 5 add prior migration experience and migration intentions as control variable. Models 3 and 6 include all socio-demographic controls as well as controlling for the wave. Models 4 and 8 restrict the sample to individuals who always answered themselves rather than a family member in wave 2 or wave 3. Age is coded as categorical variable below or above 26 years, income categories are standardized.

Table S3. Within-Person Analysis: Receiving a Magic Spell from Healer and Predicted Probability of Migration Attempt in Subsequent Waves

|  | (1) | (2) | (3) | (4) |
| --- | --- | --- | --- | --- |
| Magic spell by healer | 10.79*^***^* (0.02) | 10.14*^***^* (0.03) |  |  |
| By a sheikh |  |  | 10.62*^**^* (0.04) | 9.96*^**^* (0.04) |
| By a billewo/timowo |  |  | 22.11*^***^* (0.06) | 20.61*^**^* (0.07) |
| By sb else |  |  | 8.69*^**^* (0.03) | 7.91*^*^* (0.04) |
| Wave 3 | -14.95*^***^* (0.02) | -13.87*^***^* (0.02) | -14.39*^***^* (0.02) | -13.29*^***^* (0.02) |
| N | 1,986 | 1,778 | 1,986 | 1,778 |
| Individuals | 993 | 889 | 993 | 889 |
| Sample | Full | Respondent Only | Full | Respondent Only |
| Individual Fixed Effects | Yes | Yes | Yes | Yes |

*Notes*: Marginal effects after logistic regression with individual-level fixed effects. The DV are Migration Attempts in wave 2 or 3, coded as dummy (0,1) variables. Model 1 includes the full sample. Models 2 and 4 restrict the sample to respondents who answered all survey waves themselves. Model 3 shows the consistency of results for different types of healers. Significance levels: ^+^, *p <* 0*.*10, * *p <* 0*.*05, ** *p <* 0*.*01, *** *p <* 0*.*001. Reduced number of observations due to the fact that observations for respondents who always/never go to the healer or always/never tried to go abroad are dropped, i.e., only observations for whom we observe a change in going to the healer and a change in migration are included in the estimation.

## Table S4. Perceived Probability of Own Survival of the Migration Journey and Spiritual Practices

|  | (1) | (2) | (3) | (4) | (5) | (6) |
| --- | --- | --- | --- | --- | --- | --- |
| Magic spell by healer | 4.59*^***^* (0.74) |  | 3.24*^***^* (0.83) | 2.44*^*^* (0.90) | 2.72*^**^* (0.89) | 2.11*^*^* (0.88) |
| Wearing juju |  | 5.86*^***^* | 4.01*^***^* | 0.17 | 0.14 | 0.44 |
|  |  | (0.94) | (1.06) | (1.98) | (1.99) | (1.96) |
| Magic spell by healer x Wearing juju |  |  |  | 5.52*^*^* (2.34) | 5.82*^𝗁^* (2.35) | 5.63*^*^* (2.32) |
| Previous Migration Experience |  |  |  |  | -1.31^+^ | -3.05*^***^* |
|  |  |  |  |  | (0.75) | (0.77) |
| Migration Intention |  |  |  |  | 0.10*^***^* (0.01) | 0.08*^***^* (0.01) |
| Male |  |  |  |  |  | 10.99*^***^* (0.88) |
| Above 26 years |  |  |  |  |  | -0.03 |
|  |  |  |  |  |  | (0.90) |
| Single |  |  |  |  |  | 0.32 |
|  |  |  |  |  |  | (0.92) |
| No Children |  |  |  |  |  | 0.64 |
|  |  |  |  |  |  | (1.09) |
| Years in Education |  |  |  |  |  | 0.16^+^ |
|  |  |  |  |  |  | (0.08) |
| Std. Income |  |  |  |  |  | 2.60*^***^* (0.43) |
| Skipped meal last week |  |  |  |  |  | 4.58*^***^* (0.73) |
| Constant | 49.06*^***^* | 49.57*^***^* | 48.88*^***^* | 49.05*^***^* | 40.24*^***^* | 29.92*^***^* |
|  | (0.40) | (0.37) | (0.41) | (0.41) | (0.99) | (1.61) |
| *N* | 10109 | 10109 | 10109 | 10109 | 10109 | 10109 |
| Sample | Full | Full | Full | Full | Full | Full |

*Notes*: Significance levels: ^+^, *p <* 0*.*10, * *p <* 0*.*05, ** *p <* 0*.*01, *** *p <* 0*.*001. Results are from a linear regression model (OLS) with robust standard errors with and without socio-demographic controls. The dependent variable “perceived own survival" is re-coded to range from 0 to 1. The data are from wave 1, in which respondents were all personally interviewed. 72 respondents did not provide information on their perceived chances of survival. Model 4 includes the interaction term for going to a healer and wearing a juju. Model 5 controls for prior migration experience and migration intentions and Model 6 adds all socio-demographic controls. Age is coded as categorical variable below or above 26 years, income categories are standardized.

## Table S5. Perceived Probability of Own Survival of the Migration Journey and Migration Outcomes

Dependent variable 1: intention Dependent variable 2: plans Dependent variable 3: attempts

|  | (1) | (2) | (3) | (4) | (5) | (6) | (7) | (8) | (9) |
| --- | --- | --- | --- | --- | --- | --- | --- | --- | --- |
| Perceived own survival | 9.50*^***^* | 9.46*^***^* | 7.89*^***^* | 4.05*^**^* | 2.60^+^ | 1.05 | 3.69*^*^* | 3.75*^*^* | 3.19*^*^* |
|  | (1.02) | (1.02) | (1.00) | (1.47) | (1.34) | (1.36) | (1.54) | (1.55) | (1.57) |
| Previous Migration Experience |  | -0.59 | 1.88*^*^* |  | 8.04*^***^* | 6.60*^***^* |  | 5.43*^***^* | 4.43*^***^* |
|  |  | (0.75) | (0.74) |  | (1.06) | (1.08) |  | (1.22) | (1.26) |
| Migration Intention |  |  |  |  | 0.78*^***^* | 0.77*^***^* |  | 0.01 | 0.02 |
|  |  |  |  |  | (0.02) | (0.02) |  | (0.02) | (0.02) |
| Knowing sb who died mig. |  | 0.54 | 1.77*^*^* |  | -0.32 | -0.38 |  | -1.89 | -2.19^+^ |
|  |  | (0.80) | (0.79) |  | (1.10) | (1.11) |  | (1.25) | (1.26) |
| Male |  |  | 8.91*^***^* (0.93) |  |  | 2.86*^*^* (1.34) |  |  | 3.16*^*^* (1.35) |
| Above 26 years |  |  | -6.49*^***^* |  |  | -2.48^+^ |  |  | 3.35*^*^* |
|  |  |  | (0.87) |  |  | (1.32) |  |  | (1.48) |
| Single |  |  | 1.78^+^ |  |  | 1.02 |  |  | 0.37 |
|  |  |  | (0.91) |  |  | (1.35) |  |  | (1.49) |
| No Children |  |  | 8.54*^***^* |  |  | 2.35 |  |  | 0.94 |
|  |  |  | (1.13) |  |  | (1.68) |  |  | (1.83) |
| Years in Education |  |  | 0.19*^*^* |  |  | -0.58*^***^* |  |  | -0.23^+^ |
|  |  |  | (0.09) |  |  | (0.12) |  |  | (0.14) |
| Std. Income |  |  | -1.89*^***^* |  |  | 5.48*^***^* |  |  | 0.87 |
|  |  |  | (0.43) |  |  | (0.57) |  |  | (0.65) |
| Skipped meal last week |  |  | 7.57*^***^* |  |  | 0.55 |  |  | -1.72 |
|  |  |  | (0.68) |  |  | (1.03) |  |  | (1.14) |
| Constant | 64.56*^***^* | 64.35*^***^* | 46.10*^***^* | 57.33*^***^* | -5.07*^**^* | -1.89 | 19.58*^***^* | 18.88*^***^* | 17.18*^***^* |
|  | (0.65) | (0.91) | (1.68) | (0.93) | (1.91) | (2.77) | (0.92) | (1.65) | (2.68) |
| *N* | 10109 | 10109 | 10109 | 8949 | 8949 | 8949 | 6038 | 6038 | 6038 |
| Sample | Full | Full | Full | Full | Full | Full | Full | Full | Full |

*Notes*: Significance levels: ^+^, *p <* 0*.*10, * *p <* 0*.*05, ** *p <* 0*.*01, *** *p <* 0*.*001. Results are from a OLS Model with robust standard errors with and without socio-demographic controls. The dependent variable in Models 1-3 is *migration intentions* in wave 1, in Models 4-6 *migration plans* in wave 1, and *migration attempts* in wave 2 or 3 in Models 7-9. Perceived own survival is re-coded to range from 0 to 1. All dependent variables are coded here to range from 0 to 100. Models 3, 6 and 9 include all socio-demographic controls (Age is coded as categorical variable below or above 26 years, income categories are standardized) as well as prior migration experience, migration intentions and knowing somebody who died while attempting to migrate to Europe.

**S4. Robustness Checks**

## Table S6. Pooled Comparison: Going to the Healer and Migration Attempt in Wave 2 or 3 restricting the sample to respondents who responded personally in both waves

|  | (1) | (2) | (3) | (4) | (5) | (6) |
| --- | --- | --- | --- | --- | --- | --- |
| Magic spell by healer | 4.95*^***^* (0.01) | 4.97*^***^* (0.01) | 4.99*^***^* (0.01) |  |  |  |
| By a sheikh |  |  |  | 4.89*^***^* (0.01) | 4.89*^***^* (0.01) | 4.90*^***^* (0.01) |
| By a billewo/timowo |  |  |  | 9.55*^***^* (0.02) | 9.37*^***^* (0.02) | 9.27*^***^* (0.02) |
| By sb else |  |  |  | 4.65*^***^* (0.01) | 4.73*^***^* (0.01) | 4.80*^***^* (0.01) |
| Previous Migration Exp. |  | 2.63*^***^* (0.01) | 2.19*^***^* (0.01) |  | 2.60*^***^* (0.01) | 2.17*^***^* (0.01) |
| Migration Intention |  | 0.02^+^ | 0.02^+^ |  | 0.02^+^ | 0.02^+^ |
|  |  | (0.00) | (0.00) |  | (0.00) | (0.00) |
| Male |  |  | 2.43*^**^* (0.01) |  |  | 2.42*^**^* (0.01) |
| Above 26 years |  |  | 1.83*^*^* (0.01) |  |  | 1.82*^*^* (0.01) |
| Single |  |  | 0.87 |  |  | 0.87 |
|  |  |  | (0.01) |  |  | (0.01) |
| No Children |  |  | 1.07 |  |  | 1.07 |
|  |  |  | (0.01) |  |  | (0.01) |
| Years in Education |  |  | -0.07 |  |  | -0.07 |
|  |  |  | (0.00) |  |  | (0.00) |
| Std. Income |  |  | 0.60*^*^* (0.00) |  |  | 0.59*^*^* (0.00) |
| Skipped meal last week |  |  | -0.92 |  |  | -0.92 |
|  |  |  | (0.01) |  |  | (0.01) |
| Wave 3 | -5.75*^***^* (0.01) | -5.80*^***^* (0.01) | -5.81*^***^* (0.01) | -5.60*^***^* (0.01) | -5.66*^***^* (0.01) | -5.67*^***^* (0.01) |
| N | 11,905 | 11,904 | 11,904 | 11,905 | 11,904 | 11,904 |
| Individuals | 7,151 | 7,150 | 7,150 | 7,151 | 7,150 | 7,150 |
| Sample | Resp. Only | Resp. Only | Resp. Only | Resp. Only | Resp. Only | Resp. Only |
| Random Effects | Yes | Yes | Yes | Yes | Yes | Yes |

Notes: Marginal effects after multilevel logistic regression with intercepts varying at the level of the individual. The DV is a dummy variable taking the value 1 if the following questions were answered affirmatively, and 0 if else: “During the last 6 months, have you left your home to live in another country but returned/ Since we last talked, have you left your home to live in another country but returned?". These questions were asked in W2 and W3, respectively. The main IV recorded in all waves is “Have you recently received a magic spell or gri-gri to protect or strengthen you?". Models 1-3 pool all healer types, Models 4-6 differentiate between Sheikh (religious healer) or Billewo/Timowo (traditional healers) or somebody else. Models 2 and 5 control for prior migration experience. Models 3 and 6 further add all socio-demographic controls. Age is coded as categorical variable below or above 26 years, income categories are standardized. Robust standard errors are reported in parentheses ^+^, p < 0.10, * p < 0.05, ** p < 0.01, *** p < 0.001).

## Table S7. Pooled Between Person Comparison: Wearing a Juju and Migration Attempt in W2 or W3

|  | (1) | (2) | (3) | (4) |
| --- | --- | --- | --- | --- |
| Wearing juju | 1.46*^*^* | 0.99 | 0.91 | 0.72 |
|  | (0.01) | (0.01) | (0.01) | (0.01) |
| Previous Migration Experience |  | 2.80*^***^* (0.01) | 2.36*^***^* (0.01) | 2.26*^***^* (0.01) |
| Migration Intention |  | 0.02*^*^* | 0.02*^*^* | 0.01 |
|  |  | (0.00) | (0.00) | (0.00) |
| Male |  |  | 2.26*^**^* (0.01) | 2.58*^**^* (0.01) |
| Above 26 years |  |  | 1.47*^*^* | 1.35^+^ |
|  |  |  | (0.01) | (0.01) |
| Single |  |  | 0.86 | 0.84 |
|  |  |  | (0.01) | (0.01) |
| No Children |  |  | 0.43 | 0.56 |
|  |  |  | (0.01) | (0.01) |
| Years in Education |  |  | -0.06 | -0.05 |
|  |  |  | (0.00) | (0.00) |
| Std. Income |  |  | 0.53^+^ | 0.58*^*^* |
|  |  |  | (0.00) | (0.00) |
| Skipped meal last week |  |  | -0.71 | -0.77 |
|  |  |  | (0.01) | (0.01) |
| Wave 3 | -4.62*^***^* (0.01) | -6.57*^***^* (0.00) | -6.58*^***^* (0.00) | -6.29*^***^* (0.01) |
| N | 15,365 | 14,537 | 14,537 | 13,502 |
| Individuals | 9,275 | 9,275 | 9,275 | 8,917 |
| Sample | Full | Full | Full | Respondent Only |
| Random Effects | Yes | Yes | Yes | Yes |

*Notes*: Marginal effects after multilevel logistic regression with intercepts varying at the level of the individual. The IV is a dummy variable indicating whether the respondent wore a juju as seen by interviewer (10% of sample) or self-reported (3% of sample) in Wave 1. The DV “migration attempt" was asked in W2 and W3. Significance levels: ^+^, p < 0.10, * p < 0.05, ** p < 0.01, *** p < 0.001. Models 2 adds prior migration experience as control variable. Models 3 and 4 include all socio-demographic controls as well as controlling for the wave. Model 4 restrict the sample to individuals who answered themselves rather than a family member in wave 2 or wave 3. Age is coded as categorical variable below or above 26 years, income categories are standardized.

## Table S8. Ramadan: Going to the Healer and Migration Attempt in W2 before and during Ramadan

|  | (1) | (2) | (3) | (4) | (5) | (6) |
| --- | --- | --- | --- | --- | --- | --- |
| Magic spell by healer | 0.69*^***^* | 0.08*^***^* | 0.68*^***^* | 0.08*^***^* | 0.69*^***^* | 0.08*^***^* |
|  | (0.07) | (0.01) | (0.07) | (0.01) | (0.07) | (0.01) |
| Ramadan | 0.32*^**^* | 0.01 | 0.33*^**^* | 0.01 | 0.36*^**^* | 0.01 |
|  | (0.11) | (0.01) | (0.11) | (0.01) | (0.11) | (0.01) |
| Magic spell *×* Ramadan | -0.86*^*^* (0.33) |  | -0.87*^**^* (0.33) |  | -0.91*^**^* (0.34) |  |
| Previous Migration Experience |  |  | 0.25*^***^* (0.07) | 0.03*^***^* (0.01) | 0.20*^**^* (0.07) | 0.02*^**^* (0.01) |
| Migration Intention in W2 | 0.00^+^ | 0.00^+^ | 0.00^+^ | 0.00^+^ | 0.00^+^ | 0.00^+^ |
|  | (0.00) | (0.00) | (0.00) | (0.00) | (0.00) | (0.00) |
| Knowing sb who died mig. |  |  | -0.13^+^ | -0.02^+^ | -0.16*^*^* | -0.02*^*^* |
|  |  |  | (0.07) | (0.01) | (0.07) | (0.01) |
| Male |  |  |  |  | 0.33*^***^* (0.09) | 0.04*^***^* (0.01) |
| Above 26 years |  |  |  |  | 0.12 | 0.01 |
|  |  |  |  |  | (0.09) | (0.01) |
| Single |  |  |  |  | 0.05 | 0.01 |
|  |  |  |  |  | (0.09) | (0.01) |
| No Children |  |  |  |  | 0.02 | 0.00 |
|  |  |  |  |  | (0.11) | (0.01) |
| Years in Education |  |  |  |  | 0.00 | 0.00 |
|  |  |  |  |  | (0.01) | (0.00) |
| Std. Income |  |  |  |  | 0.04 | 0.00 |
|  |  |  |  |  | (0.03) | (0.00) |
| Skipped meal last week |  |  |  |  | -0.03 | -0.00 |
|  |  |  |  |  | (0.07) | (0.01) |
| N | 8,212 | 8,212 | 8,212 | 8,212 | 8,212 | 8,212 |

*Notes*: Marginal effects after logistic regression with intercepts varying at the level of the individual. The IV is a dummy variable indicating whether the respondent has recently received a magic spell or gri-gri to protect or strengthen them, interacted with an indicator taking the value 1 if the interview took place during Ramadan and zero if else. The DV “migration attempt" was taken from wave 2. Significance levels: ^+^, *p <* 0*.*10, * *p <* 0*.*05, ** *p <* 0*.*01, *** *p <* 0*.*001. Model 2 adds prior migration experience, knowing someone who died during the migration journey to Europe and migration intention in W2 as control variable. Model 3 includes all socio-demographic controls in addition. Age is coded as categorical variable below or above 26 years, income categories are standardized.
